# Persistent endothelial dysfunction is associated with exercise intolerance in adults despite preserved myocardial work 3 years after SARS‐CoV‐2 infection

**DOI:** 10.14814/phy2.71103

**Published:** 2026-09-29

**Authors:** Carla Cristina de Araújo Alves, Cássia da Luz Goulart, Luciana D'Ávila, Beatriz Helena Ernesto de Souza Silva, Igor Machado Alves, Brena Vitória Souza Aguiar, Claudia Conde, Ricardo Stein, Gerson Cipriano

**Affiliations:** ^1^ Laboratory of Clinical Exercise Physiology, FCE University of Brasília (UnB) Brasília DF Brazil; ^2^ Internal Medicine Department Federal University of Rio Grande Do Sul (UFRGS) Porto Alegre RS Brazil; ^3^ Exercise Cardiology Research Group Hospital de Clínicas de Porto Alegre (CardioEx‐HCPA) Porto Alegre RS Brazil

**Keywords:** brachial flow‐mediated dilation, exercise tolerance, Long COVID, myocardial work, vascular endothelium

## Abstract

Long COVID is frequently associated with persistent exercise intolerance, although the relative contributions of peripheral vascular dysfunction and myocardial function remain unclear. We investigated whether persistent endothelial dysfunction and altered myocardial work (MW) contribute to exercise intolerance approximately 3 years after SARS‐CoV‐2 infection. This case–control study included adults with Long COVID (*n* = 10) and controls (*n* = 11) who underwent cardiopulmonary exercise testing (CPET), transthoracic echocardiography with global longitudinal strain (GLS) and MW analysis, and brachial artery flow‐mediated dilation (FMD) with hyperemic shear assessment. Compared with controls, Long COVID participants exhibited lower peak V̇O_2_ (19.6 ± 2.5 vs. 24.3 ± 6.5 mL·kg^−1^·min^−1^, *p* = 0.04), percent‐predicted peak V̇O_2_ (61.1 ± 8.3 vs. 77.0 ± 14.2%, *p* = 0.006), and V̇O_2_ at first ventilatory threshold (11.0 ± 1.4 vs. 13.7 ± 3.8 mL·kg^−1^·min^−1^, *p* = 0.04). Conventional echocardiographic parameters and MW indices did not differ between groups (all *p* > 0.05). In contrast, endothelial function was impaired in the Long COVID group, with lower FMD (*p* < 0.001), peak shear rate (*p* = 0.01), AUCSR (*p* = 0.04), and AUCmax (*p* = 0.001). FMD correlated positively with exercise capacity in the Long COVID group (peak V̇O_2_: *r* = 0.68, *p* = 0.03;percent‐predicted peak V̇O_2_: *r* = 0.64, *p* = 0.04). In conclusion, 3 years after infection, Long COVID remains characterized by endothelial dysfunction and blunted hyperemic shear despite preserved MW, supporting a predominantly peripheral vascular basis for persistent exercise intolerance.

## Graphical abstract

Adults with Long COVID exhibited impaired aerobic capacity and persistent endothelial dysfunction despite preserved myocardial function approximately 3 years after SARS‐CoV‐2 infection. The association between flow‐mediated dilation (FMD) and exercise capacity suggests a potential contribution of peripheral vascular dysfunction to exercise intolerance.

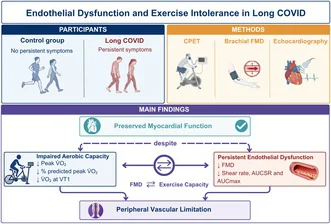

## 1 INTRODUCTION

Long COVID is a multisystem condition characterized by persistent symptoms such as fatigue, dyspnea, exercise intolerance, and cardiovascular complications that may persist for months or years after acute SARS‐CoV‐2 infection (Chuang et al., 2024; Davis et al., 2023; Rahmati et al., 2025). Affecting an estimated 10%–25% of previously infected individuals worldwide (Cornwell 3rd et al., 2025; Halas et al., 2025), Long COVID has raised growing concern regarding the mechanisms underlying long‐term cardiovascular impairment and exercise intolerance (Durstenfeld et al., 2022; Kruger et al., 2025; Singh, Joseph, et al., 2022).

Flow‐mediated dilation (FMD), a validated noninvasive measure of endothelial function (Ergul et al., 2022; Oliveira et al., 2021), is frequently impaired in affected individuals and has been related to reduced exercise capacity and adverse cardiovascular outcomes (Alexander et al., 2021; Holder et al., 2021; Ono et al., 2025). These outcomes support the concept that abnormal vascular responsiveness may contribute to persistent exercise intolerance by limiting peripheral oxygen delivery during physical activity (Faria et al., 2023; Schwendinger et al., 2024).

Although endothelial dysfunction has emerged as a potential mechanism underlying exercise intolerance in Long COVID (Ambrosino, Parrella, et al., 2022), whether subtle alterations in myocardial performance also contribute remains unclear (D'Avila et al., 2024; Ikonomidis et al., 2022). Myocardial work (MW), which integrates left ventricular deformation and afterload, can detect subtle abnormalities in myocardial function beyond conventional echocardiographic parameters (Cannata et al., 2023; Wang et al., 2021). Combined assessment of endothelial function, MW, and exercise capacity may provide a more comprehensive understanding of the mechanisms underlying exercise intolerance in Long COVID (Back et al., 2025; Jamieson et al., 2024). Whether these parameters remain associated 3 years after SARS‐CoV‐2 infection remains unknown.

To address this gap, we investigated exercise capacity, MW and endothelial function in individuals with Long COVID 3 years after SARS‐CoV‐2 infection. Using a standardized multimodal protocol, we combined cardiopulmonary exercise testing (CPET), MW assessment, and brachial artery FMD to explore the relative contributions of cardiac and peripheral vascular mechanisms to persistent exercise intolerance.

We hypothesized that individuals with Long COVID would exhibit persistent endothelial dysfunction and impaired hyperemic responsiveness associated with reduced exercise capacity despite preserved MW.

## 2 METHODS

### 2.1 Study design and ethical approval

This observational case–control study was conducted at the Laboratory of Clinical Exercise Physiology, University of Brasilia, Brazil, between 2023 and 2024. The protocol was approved by the Research Ethics Committee of the Faculty of Ceilândia, University of Brasília, Brazil (CAAE: 75183323.5.0000.8093) and conducted in accordance with the Declaration of Helsinki. Participants provided written informed consent. Reporting followed the Strengthening the Reporting of Observational Studies in Epidemiology (STROBE) guidelines (Von Elm et al., 2008). Accordingly, participants were not randomly assigned to groups but were classified according to predefined eligibility criteria.

### 2.2 Participants

Adults of both sexes who were 35 years old or older were eligible if they either (i) had no history of COVID‐19 and a documented negative RT‐PCR or antigen test; or (ii) had laboratory‐confirmed prior SARS‐CoV‐2 infection and were clinically stable. Exclusion criteria included neurological or musculoskeletal disorders, pre‐existing cardiovascular or pulmonary disease, and pregnancy.

Participants underwent clinical evaluation and were classified according to acute illness severity, based on National Institutes of Health (NIH) criteria (Gulick et al., 2024), and the presence or absence of persistent post‐COVID symptoms. Although individuals without a history of COVID‐19 were eligible as controls, all controls in the final sample had a history of asymptomatic or mild SARS‐CoV‐2 infection without persistent symptoms consistent with Long COVID. The Long COVID group included individuals with previous moderate‐to‐critical COVID‐19 and persistent symptoms.

Race was self‐reported and categorized as Brown, White, or Black. In both groups, all women included in the study were either premenopausal and using a levonorgestrel‐releasing intrauterine device with amenorrhea or postmenopausal. Participants ranged from 35 to 68 years old. Of the 24 individuals initially assessed, three were excluded, yielding a final sample of 21. The screening process, exclusions, and final group allocation are presented in the participant flowchart (Figure 1).

**FIGURE 1:**
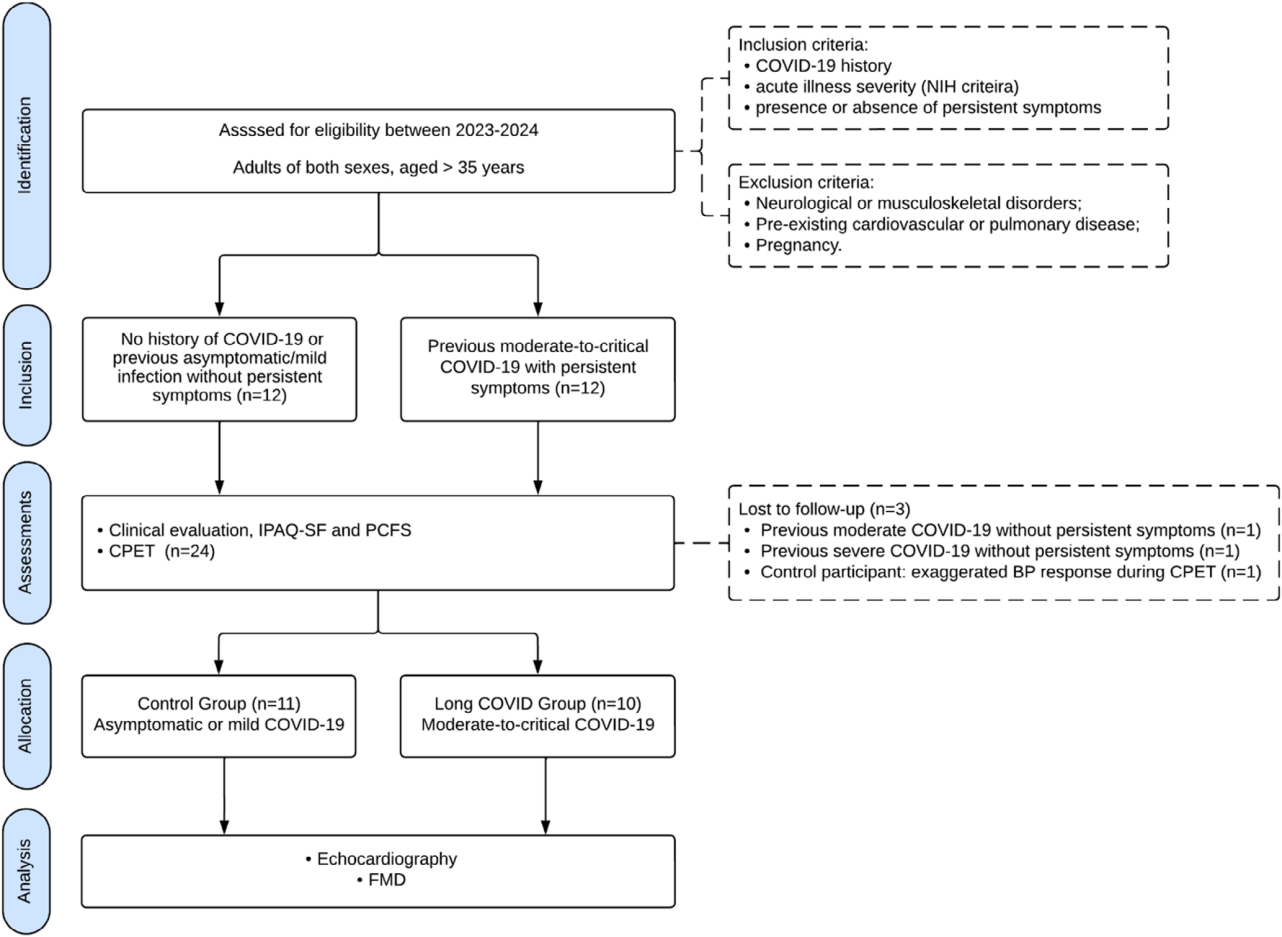
Study flowchart according to STROBE (Strengthening the Reporting of Observational Studies in Epidemiology) guidelines. CPET, cardiopulmonary exercise testing; FMD, flow‐mediated dilation; IPAQ‐SF, International Physical Activity Questionnaire–Short Form; PCFS, Post‐COVID‐19 Functional Status Scale.

### 2.3 Recruitment strategy and experimental procedures

Participants were recruited through social media advertisements and referrals from related studies. Study procedures were conducted across three visits and included clinical evaluation and CPET (V1), transthoracic echocardiography (V2), and brachial artery FMD (V3). The three‐visit design was used to minimize potential interference between assessments, particularly to prevent maximal exertion during CPET from influencing subsequent resting cardiovascular and vascular measurements. This sequential approach allowed the characterization of exercise intolerance, myocardial performance, and peripheral vascular function in individuals with Long COVID. Participants were instructed to refrain from caffeine, alcohol, and stimulant consumption for at least 12 h and to avoid strenuous physical activity for 24 h before each visit.

### 2.4 First visit

#### 2.4.1 Clinical evaluation

Clinical evaluation included demographics, anthropometrics, COVID‐19 characteristics, medical history, International Physical Activity Questionnaire—Short Form (IPAQ‐SF) (Lee et al., 2011), and post‐COVID‐19 Functional Status (PCFS) scale (De Facio et al., 2023).

#### 2.4.2 Cardiopulmonary exercise testing

After the initial clinical evaluation, cardiorespiratory fitness was assessed by CPET using a breath‐by‐breath metabolic cart (Quark CPET; COSMED, Milan, Italy), continuous 12‐lead ECG monitoring (Quark T12x; COSMED, Milan, Italy) and blood pressure (BP) monitoring (Tango M2, Suntech Medica, Morrisville, USA) (Milani et al., 2023). Before exercise, participants remained at rest for 2–3 min until a stable baseline was achieved, during which ECG, HR, and BP were assessed. These variables were monitored throughout the test and during a 3–5‐min unloaded recovery period following exercise termination (Glaab & Taube, 2022; Pritchard et al., 2021).

CPET was performed on a cycle ergometer (Corival, Lode, Groningen, The Netherlands) in a climate‐controlled environment, with temperature controlled between 16°C and 24°C and relative humidity between 30% and 60%. Tests were symptom‐limited and continued until volitional exhaustion (Milani et al., 2023). An individualized ramp‐incremental protocol was used, with linear work‐rate increments tailored to the initial clinical assessment to achieve a test duration of 8–12 min, in accordance with international recommendations (Balady et al., 2010; Herdy et al., 2016; Kabbadj et al., 2024). The system was calibrated daily following the manufacturer's instructions.

Brazilian reference equations were used to derive predicted peak V̇O_2_ and oxygen uptake efficiency slope (OUES) (Baba et al., 1996; Milani et al., 2022); predicted maximum HR (HRmax) was computed using Tanaka's formula (Tanaka et al., 2001).

### 2.5 Second visit

#### 2.5.1 Transthoracic echocardiography

Transthoracic echocardiography was performed using a Vivid S70N ultrasound system (GE Healthcare, Haifa, Israel) according to the European Association of Cardiovascular Imaging and the American Society of Echocardiography (EACVI/ASE) (Lang et al., 2015).

All analyses were performed offline in EchoPAC v203 (GE Vingmed Ultrasound, Milwaukee, WI, USA) by a single experienced investigator blinded to group allocation, following EACVI/ASE consensus (Voigt et al., 2015). GLS and MW indices, including global work index (GWI), global constructive work (GCW), global wasted work (GWW), and global work efficiency (GWE) were obtained using standard methods. NORRE reference ranges were used for interpretation (Manganaro et al., 2019; Russell et al., 2012).

### 2.6 Third visit

#### 2.6.1 Flow‐mediated dilation

Brachial artery ultrasound was performed using a high‐resolution Doppler duplex system (Philips Compact 5500G, Philips Ultrasound LLC, Bothell, WA, USA) equipped with a linear array transducer (Philips eL18‐4). The transducer was positioned longitudinally 1–3 cm proximal to the antecubital fossa. Imaging was acquired in duplex mode (B‐mode + pulsed Doppler), with the insonation angle fixed at 60°. After 20 min of supine rest, baseline diameter and mean blood velocity were recorded for 2 min. A forearm cuff was inflated to 220 mmHg for 5 min to induce ischemia. Recordings resumed 30 s before cuff release and continued for 3 min after deflation.

FMD was analyzed by a single experienced examiner (Thijssen et al., 2019; Turri‐Silva et al., 2021). FMD (%) analysis was performed in Cardiovascular Suite® using automated edge‐detection and wall‐tracking software. FMD was calculated as the percentage increase from baseline diameter to the maximum diameter occurring within 3 min after cuff deflation (Eibel et al., 2022; Turri‐Silva et al., 2021).

### 2.7 Statistical analysis

The a priori sample size calculation for the broader study protocol investigating mechanisms underlying exercise intolerance in Long COVID was based on mean arterial pressure during muscle metaboreflex assessment. Endothelial function assessed by FMD was prespecified within the same study protocol and represents a central vascular outcome of the present analysis. Using G*Power version 3.1.9.7 (Heinrich Heine University Düsseldorf, Düsseldorf, Germany; RRID: SCR_013726), with an α level of 0.05 and a statistical power of 95%, a minimum sample size of 18 participants was estimated, with an additional 10% allowance for potential attrition. For FMD, a post hoc power analysis based on the observed effect size (Cohen's *d* = 2.30), final sample size (*n* = 21), and *α* level of 0.05 was performed.

Data are presented as means ± standard deviation (SD) or as absolute frequencies (%), and 95% confidence intervals (CIs) when appropriate. Normality was assessed by the Shapiro–Wilk test. Between‐group comparisons used the unpaired Student's *t*‐test, and categorical variables were compared with Fisher's exact test. Pearson correlation analysis examined associations between variables. When appropriate, ANCOVA was performed to adjust for potential confounders, including age, sex, BMI, and comorbidities. The potential influence of baseline brachial artery diameter on FMD was additionally assessed using allometric scaling of log10‐transformed peak and baseline brachial artery diameters, with group and the group × log10‐transformed baseline diameter interaction included in the regression model (Atkinson & Batterham, 2013). Effect sizes were calculated using Cohen's d and interpreted as small (0.2–0.49), medium (0.5–0.79), and large (≥0.8) (Cohen, 1988). Significance was set at *p* < 0.05. All tests were performed using GraphPad Prism version 7.0 (GraphPad Software, California, USA; RRID: SCR_002798) and the Statistical Package for the Social Sciences (SPSS), version 27.0 (Chicago, IL, USA; RRID: SCR_002865).

## 3 RESULTS

Of the 24 individuals initially recruited, two with previous moderate and severe COVID‐19, respectively, were excluded because they did not report persistent symptoms or functional limitations consistent with Long COVID. One control was excluded because of an exaggerated BP response during CPET and was referred for cardiologic evaluation. The resulting sample comprised 21 participants, including 11 controls and 10 individuals with Long COVID (Figure 1).

### 3.1 Clinical evaluation

Baseline demographic and clinical characteristics are shown in Table 1. All control participants had a history of SARS‐CoV‐2 infection; two had asymptomatic infection and nine had mild COVID‐19, and none met the criteria for Long COVID. Compared with controls, participants with Long COVID had higher body weight (*p* = 0.02) and BMI (*p* < 0.001), a higher prevalence of depression (*p* = 0.05) and obesity (*p* = 0.002). Other variables were similar across groups.

**TABLE 1:** Baseline characteristics of Long COVID and control groups.

Variables	Control (*n* = 11)	Long COVID (*n* = 10)	*p* Value
Demographic data and anthropometric characteristics			
Female, *n* (%)	7 (64)	5 (50)	0.52
Race, *n* (%)			
Brown	3 (27)	5 (50)	0.41
White	7 (64)	3 (30)	
Black	1 (9)	2 (20)	
Age, years	47 ± 10	49 ± 9	0.69
Weight (kg)	76.9 ± 11.6	96.1 ± 22.3	0.02
Height (cm)	167.8 ± 10.2	170 ± 12.6	0.67
BMI (kg/m^2^)	27.2 ± 1.7	32.9 ± 4.5	<0.001
Time since SARS‐CoV‐2 infection, months	35 ± 12	40 ± 9	0.37
Comorbidities, *n* (%)			
Depression	0 (0)	3 (30)	0.05
Diabetes mellitus	0 (0)	2 (20)	0.11
Dyslipidemia	1 (9)	3 (30)	0.22
Hypertension	3 (27)	4 (40)	0.54
Obesity	0 (0)	7 (70)	0.002
*Acute COVID‐19 Phase*			
Symptoms, *n* (%)			
Ageusia	1 (9)	4 (40)	0.09
Anosmia	2 (18)	3 (30)	0.52
Chills	1 (9)	6 (60)	0.01
Nasal congestion	3 (27)	1 (10)	0.31
Rhinorrhea	2 (18)	2 (20)	0.91
Diarrhea	1 (9)	3 (30)	0.22
Dyspnea	1 (9)	8 (80)	0.001
Headache	4 (36)	6 (60)	0.27
Sore throat	4 (36)	2 (20)	0.40
Fatigue	2 (18)	6 (60)	0.04
Loss of appetite	0 (0)	1 (10)	0.28
Fever	1 (9)	7 (70)	0.004
Myalgia	0 (0)	1 (10)	0.28
Cough	3 (27)	4 (40)	0.53
Clinical Management, *n* (%)			
Hospitalization		6 (60)	—
Oxygen therapy		6 (60)	—
Mechanical ventilation		1 (10)	—

*Note*: Continuous data are presented as mean ± SD; categorical data as absolute and relative values *n* (%). Comparisons used Student's *t*‐test (continuous) and Fisher's exact test (categorical).

Abbreviation: BMI, body mass index.

During the acute phase, the Long COVID group more frequently reported chills, dyspnea, fatigue, and fever (all *p* ≤ 0.04). No controls required hospitalization, whereas in the Long COVID 60% were hospitalized, 60% received oxygen therapy, and 10% required mechanical ventilation; disease severity was moderate in 20%, severe in 70%, and critical in 10%.

Long COVID participants reported persistent symptoms, including fatigue (60%), exercise intolerance (50%), dyspnea (40%), and headache (20%). Vaccination status and IPAQ classification did not differ between groups, although weekly MET‐minutes were lower in Long COVID (*p* = 0.02). PCFS scores also indicated greater functional limitation in Long COVID (*p* < 0.001; Table 2).

**TABLE 2:** Symptoms, vaccination status, physical activity, and functional status in Long COVID and controls.

Variables	Control (*n* = 11)	Long COVID (*n* = 10)	*p* Value
Persistent symptoms, *n* (%)			
Dyspnea		4 (40)	—
Headache		2 (20)	—
Fatigue		6 (60)	—
Exercise intolerance		5 (50)	—
Vaccination (number of doses), *n* (%)			
None	1 (9)	1 (10)	
2	1 (9)	0	
3 or more	9 (82)	9 (90)	0.28
Physical Activity level—IPAQ‐SF, *n* (%)			
Low	1 (9)	3 (30)	
Moderate	6 (55)	7 (70)	0.16
High	4 (36)	0	
MET (min/week)	4352 ± 3833	1425 ± 776	0.02
Degree of functional limitation—PCFS, *n* (%)			
0	10 (91)	2 (20)	
1	1 (9)	0	
2	0	4 (40)	<0.001
3	0	4 (40)	
4	0	0	

*Note*: Continuous data are presented as mean ± SD; categorical data as absolute and relative values *n* (%). Comparisons used Student's *t*‐test (continuous) and Fisher's exact test (categorical).

Abbreviations: IPAQ‐SF, International Physical Activity Questionnaire short form; PCFS, Post‐COVID‐19 Functional Status Scale.

#### 3.1.1 Cardiopulmonary exercise testing

Participants with Long COVID demonstrated significantly reduced exercise capacity compared with controls, with lower peak V̇O_2_ (Cohen's *d* = 0.82; *p =* 0.04), predicted peak V̇O_2_ (Cohen's *d* = 1.35; *p =* 0.006), and V̇O_2_ at first ventilatory threshold [VT_1_ (Cohen's *d* = 0.93; *p =* 0.04)] as detailed in Table S1 and illustrated in Figure 2a–c. No additional between‐group differences were observed for the remaining CPET variables (Table S1).

**FIGURE 2:**
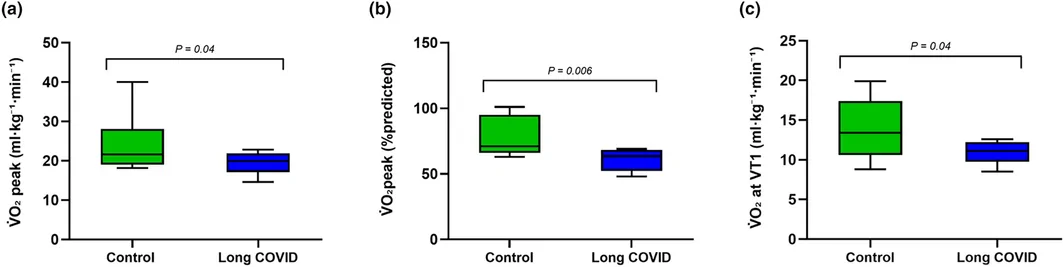
Group comparisons of cardiopulmonary exercise testing (CPET) outcomes in control (*n* = 11) and Long COVID (*n* = 10) groups. (a) Relative peak oxygen uptake (peak V̇O_2_, mL·kg^−1^·min^−1^; *p* = 0.04); (b) Predicted peak V̇O_2_ (%; *p* = 0.006); (c) Oxygen uptake at the first ventilatory threshold (V̇O_2_ at VT_1_, mL·kg^−1^·min^−1^; *p* = 0.04). Boxes represent the interquartile range (25th–75th percentiles), center lines indicate the median, and whiskers represent the minimum and maximum values. Comparisons used Student's *t*‐test.

#### 3.1.2 Transthoracic echocardiography

No significant between‐group differences were observed in echocardiographic parameters, including conventional indices, GLS, and MW (all *p* > 0.05; Table 3). Figure S1 shows a representative bull's‐eye (polar) map of LV peak systolic strain and MW.

**TABLE 3:** Baseline echocardiographic profile of Long COVID and control groups.

Variables	Control (*n* = 11)	Long COVID (*n* = 10)	Mean difference (95% CI)	Cohen's *d*	*p* Value
LAV (ml/m^2^)	45 (± 11)	46 (± 12)	1 (−9, 11)	0.23	0.61
RVs (cm/s)	14 (± 2)	14 (± 3)	0 (−3, 2)	0.17	0.71
TAPSE (mm)	22 (± 2)	21 (± 2)	−1 (−3, 1)	0.55	0.23
LVEDV (ml)	86 (± 31)	77 (± 18)	−8 (−31, 15)	0.36	0.45
LVESV (ml)	26 (± 11)	24 (± 6)	−2 (−10, 6)	0.26	0.58
LVEF (Simpson, %)	69 (± 2)	69 (± 2)	−1 (−3, 1)	0.37	0.43
LV GLS (%)	21.1 (± 1.7)	20 (± 2.2)	−1.2 (−3, 0.6)	0.59	0.202
GWI (mmHg%)	2371 (± 263)	2316 (± 469)	−55 (−419, 308)	0.12	0.797
GCW (mmHg%)	2419 (± 278)	2395 (± 466)	−24 (−387, 340)	0.00	0.995
GWW (mmHg%)	84 (± 35)	115 (± 45)	31 (−7, 69)	0.73	0.119
GWE (%)	96 (± 2)	95 (± 2)	−2 (−3, 0)	0.81	0.085

*Note*: Data are mean ± SD. Group comparisons used Student's *t*‐test, and effect sizes are reported as Cohen's *d*.

Abbreviations: GCW, global constructive work; GLS, global longitudinal strain; GWE, global work efficiency; GWI, global work index; GWW, Global wasted work; LAV, Left atrial volume; LV, left ventricular; LVEDV, left ventricular end‐diastolic volume; LVEF, left ventricular ejection fraction; LVESV, left ventricular end‐systolic volume; RVs, Right ventricular systole; TAPSE, tricuspid annular plane systolic excursion.

GWI was similar between groups (*p* > 0.05; Figure S2a and Table 3). Group‐specific analyses revealed distinct patterns of association between MW indices and ventilatory responses during exercise (Table S2). In the Long COVID group, GWI was inversely correlated with peak minute ventilation (V̇Epeak; *r* = −0.72, *R* ^2^ = 0.51, *p* = 0.02), the V̇E/V̇CO_2_ slope at peak exercise (*r* = −0.69, *R* ^2^ = 0.48, *p* = 0.02), and the ventilatory equivalent for oxygen at VT_1_ (*r* = −0.68, *R* ^2^ = 0.46, *p* = 0.03) and VT_2_ (*r* = −0.68, *R* ^2^ = 0.46, *p* = 0.03), Figure S2b–e. In addition, GWI showed a strong inverse association with BMI in the Long COVID group (*r* = −0.80, *R* ^2^ = 0.64, *p* = 0.005), whereas no significant relationship was observed in controls (*r* = −0.20, *p* = 0.55), Figure S2f.

GCW values were similar between groups (*p* > 0.05; Figure S3a and Table 3). However, within‐group analyses demonstrated that GCW was inversely correlated with ventilatory inefficiency in the Long COVID group, including the peak V̇E/V̇CO_2_ slope (*r* = −0.72, *R* ^2^ = 0.52, *p* = 0.01) and the V̇E/V̇O_2_ at VT_1_ (*r* = −0.70, *p* = 0.02) and VT_2_ (*r* = −0.70, *p* = 0.02), as demonstrated in Figure S3b–d. In controls, the only significant association observed was an inverse correlation between GCW and the peak V̇E/V̇CO_2_ slope (*r* = −0.64, *R* ^2^ = 0.41, *p* = 0.03), Figure S3b.

#### 3.1.3 Flow‐mediated dilation

Compared with controls, the Long COVID group showed markedly lower FMD (Cohen's *d* = 2.30; *p <* 0.001; Table S3 and Figure 3) and a reduced peak shear‐rate stimulus (Cohen's *d* = 1.15; *p* = 0.01; Table S3 and Figure 4a). In addition, the area‐under‐the‐curve shear‐rate stimulus (AUCSR), calculated over the hyperemic period, was lower (Cohen's *d* = 0.93; *p* = 0.04; Table S3 and Figure 4b), and the AUC up to maximal vasodilation (AUC max) was also substantially reduced in Long COVID compared with controls (Cohen's *d* = 1.65; *p* = 0.001; Table S3 and Figure 4c).

**FIGURE 3:**
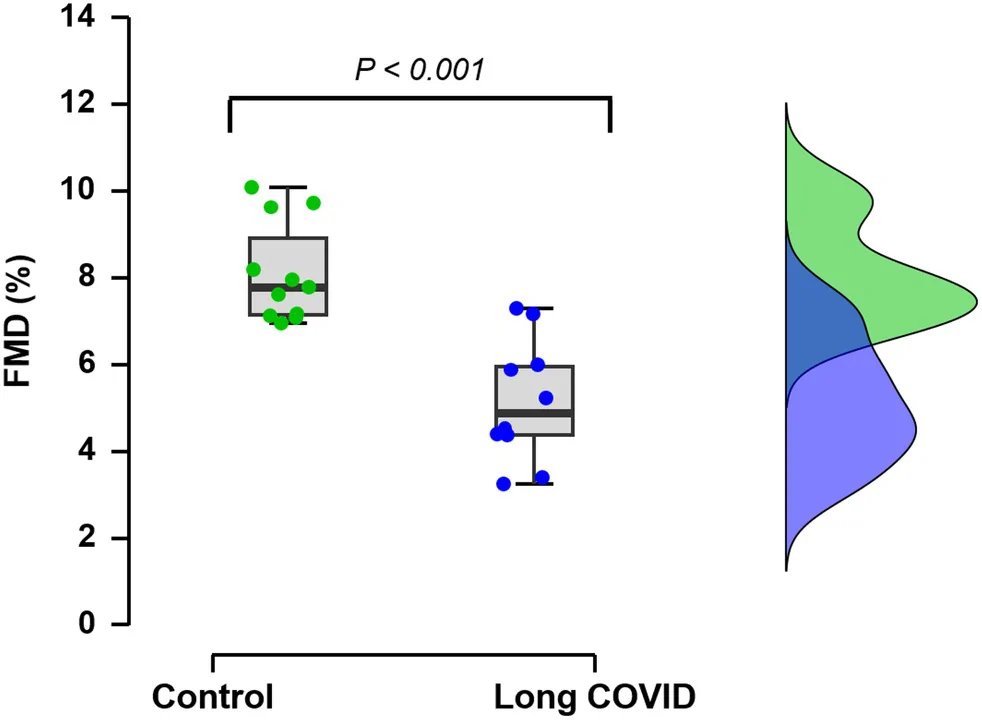
Comparison of endothelial function in control (*n* = 11) and Long COVID (*n* = 10) groups assessed by flow‐mediated dilation [FMD (%); *p* < 0.001]. Data are presented as raincloud plots, combining jittered individual observations, boxplots, and density distributions. Control and Long COVID groups are represented in green and blue, respectively. Boxplots indicate the median and interquartile range, whereas density plots illustrate the distribution of FMD values within each group. Comparisons were performed using Student's *t*‐test.

**FIGURE 4:**
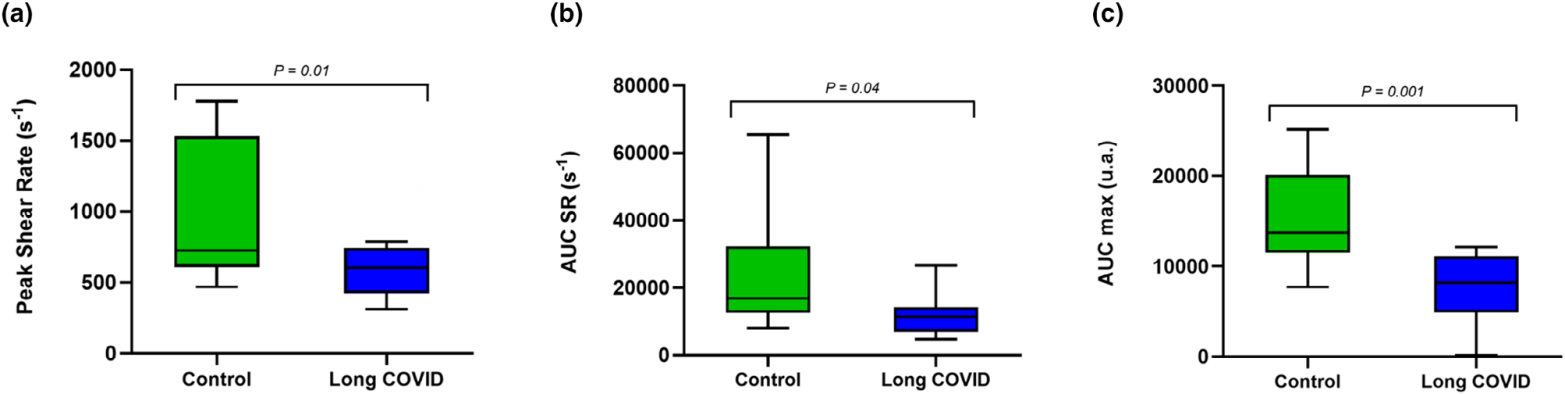
Group comparisons of hyperemic shear parameters in control (*n* = 11) and Long COVID (*n* = 10) groups. (a) Peak shear rate (s^−1^; *p* = 0.01); (b) AUCSR, area under the curve of shear rate (s^−1^; *p* = 0.04); (c) AUC max, area under the curve up to maximum vasodilation (u.a.; *p* = 0.001). Boxes represent the interquartile range (25th–75th percentiles), center lines indicate the median, and whiskers represent the minimum and maximum values. Comparisons used Student's *t*‐test.

Allometric analysis showed no significant group × log10‐transformed baseline diameter interaction (*p* = 0.74). Using a common‐slope model, the scaling exponent was 0.98 (95% CI, 0.95–1.02), and the between‐group difference remained significant after adjustment for log10‐transformed baseline diameter (*p* = 0.02).

Resting and post‐deflation diameters, recovery, FMD expressed in millimeters, and shear rate at rest showed no differences between groups (all *p* > 0.05; Table S3). A representative FMD assessment (B‐mode images, Doppler traces, and time courses of diameter and shear rate) from a control and a Long COVID participant is shown in Figure 5.

**FIGURE 5:**
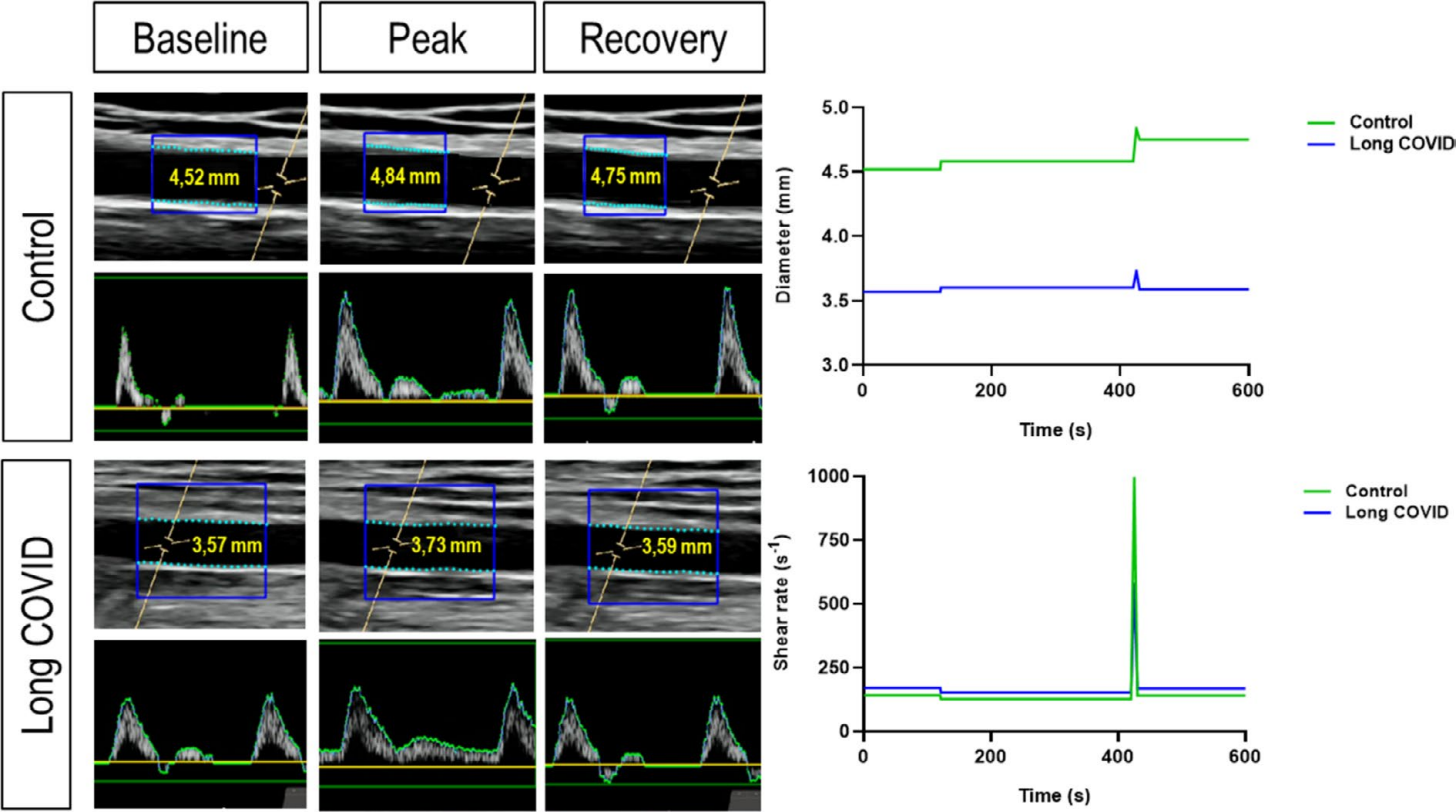
Representative brachial artery flow‐mediated dilation (FMD) assessment in a control and a long COVID participant. Left panels show B‐mode ultrasound images with diameter measurements at baseline, peak dilation (maximum), and recovery, along with the corresponding Doppler traces. Right panels depict the time courses of arterial diameter (mm) and shear rate (s^−1^) throughout the protocol, illustrating the transient hyperemic shear‐rate stimulus and the subsequent vasodilatory response. Lines: Control (green) and Long COVID (blue).

#### 3.1.4 Associations of flow‐mediated dilation with exercise capacity, body mass index, and myocardial work

Correlation analyses were then performed separately within each group. In the Long COVID group, FMD was significantly and positively correlated with peak V̇O_2_ (*r* = 0.68; *R* ^2^ = 0.46; *p* = 0.03) and predicted peak V̇O_2_ (*r* = 0.64; *R* ^2^ = 0.40; *p* = 0.04; Figure 6a,b). In contrast, these associations were not observed in controls.

**FIGURE 6:**
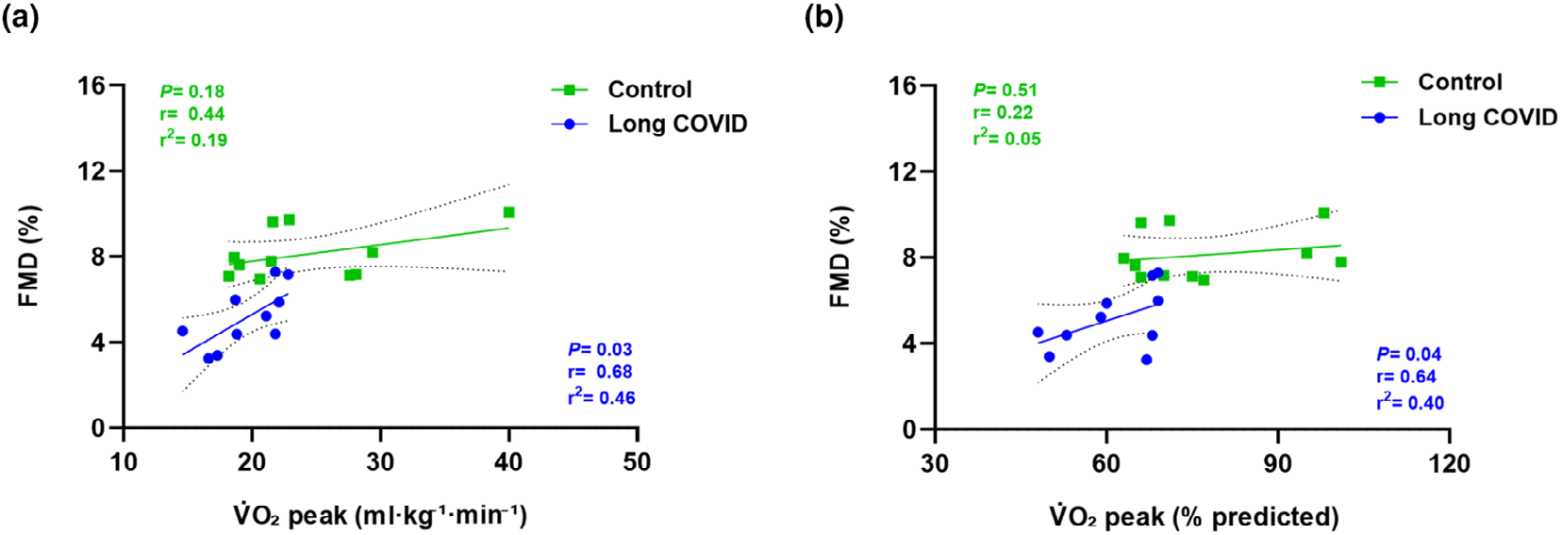
Associations between endothelial function and exercise capacity in control (*n* = 11) and Long COVID (*n* = 10) groups. (a) Correlation between flow‐mediated dilation [FMD (%)] and peak oxygen uptake (peak V̇O_2_, mL·kg^−1^·min^−1^). (b) Correlation between FMD (%) and predicted peak oxygen uptake (% predicted peak V̇O_2_). Long COVID and control groups are shown in blue and green, respectively. Each point represents one participant. Solid lines indicate linear regression and dotted lines represent the 95% confidence interval. Pearson's *r*, *R* ^2^, and *p* values are presented in panels a and b.

Additionally, in the control group, FMD was significantly and inversely correlated with GWW (*r* = −0.72; *R* ^*2*^ = 0.51; *p* = 0.01), whereas no significant association between FMD and GWW was observed in the Long COVID group (*r* = 0.46; *R* ^*2*^ = 0.21; *p* = 0.18; Figure S4A). Moreover, no significant associations between FMD and BMI were found in either group (Figure S4B).

## 4 DISCUSSION

Despite growing recognition of the long‐term cardiovascular consequences of SARS‐CoV‐2 infection (Davis et al., 2023; Xie et al., 2022; Zhang et al., 2025), studies integrating vascular function, MW, and exercise capacity remain scarce (Schwendinger et al., 2024; Serviente et al., 2022). In the present study, Long COVID participants exhibited persistent endothelial dysfunction and reduced aerobic capacity approximately 3 years after infection, whereas conventional echocardiographic parameters and MW indices remained preserved. Importantly, endothelial function was correlated positively with exercise capacity only in the Long COVID group, suggesting a potential link between peripheral vascular dysfunction and long‐term exercise limitation (Ambrosino, Parrella, et al., 2022; Back et al., 2025).

Endothelial dysfunction has emerged as a central pathophysiological feature of Long COVID (Kruger et al., 2025; Stojanovic et al., 2025; Wu et al., 2024). Persistent endothelial injury after SARS‐CoV‐2 infection has been associated with impaired nitric oxide bioavailability, chronic inflammation, oxidative stress, and microvascular dysfunction, all of which may compromise vasodilatory capacity (Amraei & Rahimi, 2020; Wu et al., 2024). In our cohort, lower FMD, accompanied by reduced shear rate, AUCSR, and AUCmax indicated impaired conduit artery endothelial responsiveness and blunted hyperemic signaling (Ergul et al., 2022; Kruger et al., 2025), in accordance with previous reports of persistent endothelial impairment and vascular abnormalities after COVID‐19 (Ambrosino, Sanduzzi Zamparelli, et al., 2022; Ikonomidis et al., 2022; Oliveira et al., 2021).

Importantly, the concomitant reduction in shear‐related indices suggests that endothelial dysfunction in Long COVID involves not only impaired vasodilatory responsiveness but also a diminished hyperemic stimulus which may contribute to impaired peripheral oxygen delivery during exercise, in agreement with previous studies (Ambrosino, Parrella, et al., 2022; Back et al., 2025; Jothi et al., 2025). Collectively, these vascular abnormalities provide a plausible physiological basis for the persistent reduction in exercise capacity observed in Long COVID (Faria et al., 2023; Schwendinger et al., 2024).

During the first year after SARS‐CoV‐2 infection, reduced exercise capacity has been more frequently attributed to circulatory than ventilatory constraints (Durstenfeld et al., 2022; Singh, Joseph, et al., 2022), a physiologically plausible scenario given that vascular dysfunction can limit oxygen delivery to working skeletal muscle and thereby impair aerobic performance (Longobardi et al., 2022; Serviente et al., 2022). Consistent with this framework, participants with Long COVID exhibited lower aerobic capacity, whereas resting cardiac structure and systolic function were preserved in our sample, suggesting that central cardiac dysfunction is unlikely to be the primary determinant of exercise intolerance (Schwendinger et al., 2024; Singh, Joseph, et al., 2022; Tryfonos et al., 2024).

A previous study reported the coexistence of endothelial dysfunction and reduced exercise capacity approximately 2 years after SARS‐CoV‐2 infection (Ono et al., 2025). By extending the follow‐up to approximately 3 years, we show that these abnormalities persist well beyond the second year after the acute illness. Long COVID participants exhibited lower relative peak V̇O_2_ and V̇O_2_ at VT_1_ than controls, indicating persistent impairment of both maximal and submaximal aerobic capacity. Our observations extend previous CPET studies documenting sustained exercise intolerance following SARS‐CoV‐2 infection (Longobardi et al., 2022; Salmam et al., 2025; Tryfonos et al., 2024).

Moreover, FMD was positively correlated with peak V̇O_2_ and predicted peak V̇O_2_ exclusively in the Long COVID group (Back et al., 2025; Ono et al., 2025), supporting a link between vascular function and aerobic performance in these individuals (Ambrosino, Parrella, et al., 2022; Back et al., 2025). In contrast, only controls exhibited an inverse association between FMD and GWW, indicating that better endothelial function was accompanied by lower myocardial inefficiency (Heiss et al., 2023; Ikonomidis et al., 2022). To our knowledge, no previous studies have specifically investigated the association between FMD and GWW in healthy adults.

Beyond peripheral vascular dysfunction, accumulating evidence suggests that SARS‐CoV‐2 infection also affects MW, potentially through both direct myocardial injury and indirect mechanisms, including systemic inflammation, endothelial dysfunction, and microvascular thrombosis (Babapoor‐Farrokhran et al., 2020; Li et al., 2025; Rusu et al., 2022). In some individuals, MW indices such as GWI and GWE are reduced after infection, even following mild‐to‐moderate illness, and may coincide with elevated cardiac biomarkers (Dankowski et al., 2024; Minhas et al., 2021; Olsen et al., 2022).

Although conventional echocardiographic parameters often remain preserved, advanced echocardiography can detect subclinical myocardial dysfunction that persists for months, potentially driven by oxidative stress, endothelial injury, and microvascular abnormalities. These alterations have been linked to persistent symptoms and increased cardiovascular risk (Cannata et al., 2023; Ikonomidis et al., 2022; Shchendrygina et al., 2025), highlighting the potential value of MW assessment for post‐COVID cardiovascular phenotyping and risk stratification (Bavishi et al., 2020; D'Avila et al., 2024).

To further explore the contribution of myocardial function, we examined how MW relates to respiratory responses during exercise. Dyspnea was common during both the acute phase and follow‐up in the Long COVID group (Piamonti et al., 2024; Weberling et al., 2025). While mean GWI values were similar between groups, lower GWI in Long COVID was related to greater ventilatory drive and poorer ventilatory efficiency, whereas more preserved GWI tracked with a more economical breathing pattern (Ahn et al., 2024). These findings are consistent with a recent study showing that, despite preserved EF and normal conventional echocardiographic parameters, Long COVID patients with persistent dyspnea exhibit lower GWI and GCW, both independently linked to symptoms (Luchian et al., 2022). CPET studies further demonstrate impaired ventilatory efficiency and reduced exercise capacity in these individuals, consistent with a ventilation/perfusion mismatch or hyperventilation syndrome (Ambrosino, Parrella, et al., 2022; Milani et al., 2023; Singh, Joseph, et al., 2022).

Our findings also highlight the interaction between metabolic status and myocardial mechanics in Long COVID. Participants with Long COVID had higher BMI, which was inversely associated with GWI within this group, consistent with previous evidence linking obesity and metabolic dysfunction to cardiovascular abnormalities in Long COVID (Ergul et al., 2022; Singh, Rathore, et al., 2022; Tudoran et al., 2022; Tudoran et al., 2023). Conversely, BMI was not associated with FMD in either group (Santos‐De‐Araujo et al., 2026), suggesting that the relationship between adiposity and MW abnormalities may not be fully captured by conduit artery endothelial dysfunction (Gasparkova et al., 2026; Ikonomidis et al., 2022; Zhao et al., 2025). Importantly, the between‐group differences remained significant after adjustment for age, sex, weight, height, BMI, and comorbidities.

### 4.1 Limitations and clinical implications

This study has several limitations that should be considered when interpreting the outcomes. First, its observational, cross‐sectional, single‐center design precludes causal inference and may limit the generalizability of the findings. Despite the modest sample size, an a priori sample size calculation was performed, and several key between‐group differences were accompanied by large effect sizes. Nevertheless, the findings should be considered hypothesis‐generating and require confirmation in larger, multicenter studies (Perez‐Guerrero et al., 2024; Wang & Cheng, 2020).

Second, because the Long COVID cohort consisted predominantly of individuals with previous moderate‐to‐critical SARS‐CoV‐2 infection and persistent symptoms, our findings may not be generalizable to the broader spectrum of post‐COVID conditions, particularly individuals with asymptomatic or mild infection. In addition, all control participants had a history of asymptomatic or mild SARS‐CoV‐2 infection. Although none met the criteria for Long COVID, subtle long‐term consequences of infection cannot be completely excluded, which may have influenced between‐group comparisons.

Third, although CPET provided objective physiological characterization of exercise capacity, it served to characterize rather than define the study population (Contreras et al., 2024; Durstenfeld et al., 2022; Salmam et al., 2025). Furthermore, exercise capacity was lower in the Long COVID participants; however, the overlap in exercise performance between groups suggests that the observed endothelial dysfunction cannot be explained solely by differences in cardiorespiratory fitness (Ambrosino, Parrella, et al., 2022; Back et al., 2025; Santos‐De‐Araujo et al., 2026).

Fourth, the cohort was predominantly composed of individuals with overweight or obesity, and it remains uncertain whether non‐obese individuals would exhibit similar vascular, cardiac, and exercise responses (Lacavalerie et al., 2022; Sanhueza et al., 2023). In addition, the study was not powered to detect sex‐ or race‐related differences, which limits further interpretation of potential subgroup effects (Akbar et al., 2025; Peter et al., 2025; Sylvester et al., 2022).

Fifth, the exploratory analyses involving MW were not intended to establish causal or mechanistic relationships and should therefore be interpreted cautiously. These associations require confirmation in larger cohorts (Luijken et al., 2022; Roberts et al., 2023). Finally, prior or ongoing cardiopulmonary or metabolic rehabilitation was not systematically assessed, and its possible influence on endothelial function, MW, and exercise capacity cannot be excluded (Choi et al., 2025; Cornwell 3rd et al., 2025; McDowell et al., 2025).

Despite these limitations, the present study has important mechanistic and clinical implications. By integrating endothelial function, hyperemic shear, MW, and exercise physiology approximately 3 years after SARS‐CoV‐2 infection, this study extends previous clinical investigations beyond the first 2 years after infection (Ambrosino, Parrella, et al., 2022; Durstenfeld et al., 2023; Faria et al., 2023; Ono et al., 2025). This integrated assessment provides further insight into the vascular abnormalities associated with persistent exercise intolerance and supports a more comprehensive cardiovascular evaluation of individuals with long‐term post‐COVID symptoms (D'Avila et al., 2024; Ikonomidis et al., 2022; Jamieson et al., 2024; Serviente et al., 2022).

In this context, brachial FMD, interpreted alongside the hyperemic shear stimulus, may provide a noninvasive index of endothelial health that complements CPET in distinguishing peripheral from central contributors to reduced exercise capacity (Ikonomidis et al., 2022; Schwendinger et al., 2024). Moreover, the coexistence of endothelial dysfunction and reduced aerobic capacity despite preserved conventional cardiac function and MW underscores the importance of considering peripheral vascular abnormalities when evaluating persistent exercise intolerance in Long COVID (Ikonomidis et al., 2022; Ono et al., 2025; Schwendinger et al., 2024; Singh, Joseph, et al., 2022).

These observations reinforce the rationale for comprehensive, cardiometabolic risk–oriented management in Long COVID, including optimization of BP, lipids, glycemic control, and body weight, alongside structured exercise‐based rehabilitation (Chuang et al., 2024; De Lorenzo et al., 2021; Khunti et al., 2021). Future longitudinal and interventional studies involving larger and more diverse populations are needed to determine whether improvements in endothelial function and MW translate into better exercise tolerance and reduced long‐term cardiovascular risk in this growing patient population.

## 5 CONCLUSIONS

Long COVID was associated with persistent endothelial dysfunction and blunted hyperemic shear approximately 3 years after acute SARS‐CoV‐2 infection, despite preserved cardiac structure and myocardial function. Overall, the present data support a predominantly peripheral vascular mechanism underlying persistent exercise limitation. Although the integrated assessment of endothelial function, myocardial function, and exercise capacity may improve the cardiovascular phenotyping of Long COVID, its clinical implications remain uncertain. Well‐designed longitudinal and interventional studies are required to determine the prognostic significance of persistent endothelial dysfunction and whether improving vascular function can enhance functional recovery in this population.

## AUTHOR CONTRIBUTIONS

**Carla Cristina de Araújo Alves:** Conceptualization; data curation; formal analysis; investigation; methodology; project administration; visualization. **Cássia da Luz Goulart:** Data curation; formal analysis; investigation; methodology; supervision; validation. **Luciana D'Ávila:** Data curation; formal analysis; investigation; validation; visualization. **Beatriz Helena Ernesto de Souza Silva:** Data curation; formal analysis; investigation. **Igor Machado Alves:** Data curation; formal analysis; investigation; visualization. **Brena Vitória Souza Aguiar:** Data curation; formal analysis; investigation. **Claudia Conde:** Data curation; formal analysis; investigation. **Ricardo Stein:** Data curation; formal analysis; funding acquisition; resources; supervision; validation. **Gerson Cipriano Jr.:** Conceptualization; formal analysis; funding acquisition; methodology; project administration; resources; supervision; validation.

## FUNDING INFORMATION

This research received no specific grant from any funding agency in the public, commercial, or not‐for‐profit sectors.

## CONFLICT OF INTEREST STATEMENT

The authors report no financial or personal relationships with other people or organizations that could inappropriately influence or bias their work. The authors report no conflict of interest.

## ETHICS STATEMENT

The study protocol was approved by the Research Ethics Committee of the Faculty of Ceilândia, University of Brasília, Brazil (CAAE: 75183323.5.0000.8093) and conducted in accordance with the Declaration of Helsinki. All participants provided written informed consent.

## Supporting information


Appendix S1.

## Data Availability

Data supporting the findings are available from the corresponding author upon reasonable request.
