# Supplementary material for: Persistent endothelial dysfunction is associated with exercise intolerance in adults despite preserved myocardial work 3 years after SARS‐CoV‐2 infection
Source: Physiol Rep. 2026 Sep 29;14(19):e71103. doi: 10.14814/phy2.71103 (PMC13623666; doi:10.14814/phy2.71103)
Supplement: Supplementary file 1 [file PHY2-14-e71103-s001.docx]

# Supplemental Material

**Supplemental Fig. S1.** Representative image of a 44-year-old patient with Long COVID who underwent echocardiographic assessment with speckle-tracking. Bull’s-eye plots (polar maps) of peak systolic strain (left) and left ventricular myocardial work index (right) are shown for all 17 segments. GLS: −17%; PSD: 66 ms; HR (apical 4-chamber): 67 bpm; BP: 130/80 mmHg; GWI: 1924 mmHg%; GWE: 91%. GLS: global longitudinal strain; PSD: peak strain dispersion; HR: heart rate; BP: blood pressure; GWI: global work index; GWE: global work efficiency.

**Supplemental Fig. S2.** Left ventricular (LV) global work index (GWI) and its associations with ventilatory responses during cardiopulmonary exercise testing and body mass index in control (n = 11) and Long COVID (n = 10) groups. A - Mean GWI (mmHg%) in control and Long COVID groups. B - Correlation between GWI and peak minute ventilation (V̇E, L/min). C - Correlation between GWI and the V̇E/V̇CO₂ slope. D - Correlation between GWI and the ventilatory equivalent for oxygen (V̇E/V̇O₂) at the first ventilatory threshold (VT₁). E - Correlation between GWI and V̇E/V̇O₂ at the second ventilatory threshold (VT₂). F - Correlation between GWI and body mass index (BMI, kg/m²). Boxes represent the interquartile range (25th–75th percentiles), center lines indicate the median, and whiskers represent the minimum and maximum values. Comparisons used Student’s t-test. Each point represents one participant. Solid lines indicate linear regression and dotted lines represent the 95% confidence interval. Pearson’s r, R², and *p* values are presented in panels B–F.

**Supplemental Fig. S3.** Left ventricular (LV) global constructive work (GCW) and its associations with ventilatory responses during cardiopulmonary exercise testing in control (n = 11) and Long COVID (n = 10) groups. A - Mean GCW (mmHg%) in control and Long COVID groups. B - Correlation between GCW and the V̇E/V̇CO₂ slope. C - Correlation between GCW and the ventilatory equivalent for oxygen (V̇E/V̇O₂) at the first ventilatory threshold (VT₁). D - Correlation between GCW and V̇E/V̇O₂ at the second ventilatory threshold (VT₂). Boxes represent the interquartile range (25th–75th percentiles), center lines indicate the median, and whiskers represent the minimum and maximum values. Comparisons used Student’s t-test. Each point represents one participant. Solid lines indicate linear regression and dotted lines represent the 95% confidence interval. Pearson’s r, R², and *p* values are presented in panels B–D.

**Supplemental Fig. S4.** Associations of endothelial function with global wasted work and body mass index in control (n = 11) and Long COVID (n = 10) groups. A - Correlation between flow-mediated dilation [FMD (%)] and left ventricular global wasted work (GWW, mmHg%). B - Correlation between FMD (%) and body mass index (BMI, kg/m²). Long COVID and control groups are shown in blue and green, respectively. Each point represents one participant. Solid lines indicate linear regression and dotted lines represent the 95% confidence interval. Pearson’s r, R², and *p* values are presented in panels A-B.

**Supplemental Table S1.** Cardiopulmonary Exercise Testing Parameters in Long COVID and Control Groups

| **Variables** | **Control (n=11)** | **Long COVID (n=10)** | **Mean Difference**  **(95% CI)** | **Cohen’s *d*** | ***p-*value** |
| --- | --- | --- | --- | --- | --- |
| Exercise duration (min) | 12.43 (± 2.38) | 11.33 (± 2.57) | -1.1 ( -3.38, 1.18) | 0.44 | 0.32 |
| RER | 1.13 (± 0.06) | 1.12 (± 0.06) | -0.01 ( -0.07, 0.05) | 0.16 | 0.71 |
| V̇E (L/min) | 79.6 (± 28.5) | 83.6 (± 31) | 4 ( -23.4, 31.4) | 0.14 | 0.76 |
| OUES | 2113 (± 608) | 2096 (± 620) | -18 ( -581, 546) | 0.03 | 0.94 |
| OUES (% predicted) | 87.5 (± 21.5) | 76.2 (± 15.6) | -11.4 ( -28.5, 5.7) | 0.60 | 0.18 |
| Peak |  |  |  |  |  |
| V̇O₂ (L/min) | 1.9 (± 0.64) | 1.97 (± 0.61) | 0.06 ( -0.51, 0.64) | 0.10 | 0.82 |
| V̇O₂ (ml·kg⁻¹·min⁻¹) | 24.3 (± 6.5) | 19.6 (± 2.5) | -4.7 ( -9, 0.1) | 0.82 | 0.04 |
| V̇O₂ (% predicted) | 77 (± 14.2) | 61.1 (± 8.3) | -15.9 ( -26.6, -5.3) | 1.35 | 0.006 |
| VVM (L/min) | 129 (± 41) | 115 (± 25) | -14 ( -48, 19) | 0.41 | 0.50 |
| V̇E/V̇CO₂ slope | 35 (± 4) | 36 (± 6) | 1 ( -3, 6) | 0.29 | 0.52 |
| HR (bpm) | 153.36 (± 19.61) | 150.2 (± 19.59) | -3.16 ( -21.16, 14.83) | 0.16 | 0.71 |
| WR (W) | 142 (± 61) | 148 (± 46) | 6 ( -44, 55) | 0.11 | 0.81 |
| HR (% predicted) | 90.5 (± 8.6) | 89.1 (± 10.2) | -1.4 ( -10.2, 7.3) | 0.15 | 0.72 |
| O2 pulse (mL.beat⁻¹) | 12.5 (± 4.2) | 13.6 (± 4.6) | 1.1 ( -3, 5.1) | 0.24 | 0.59 |
| VT1 |  |  |  |  |  |
| Time (min) | 5.9 (± 1.7) | 6.2 (± 1.7) | 0.3 ( -1.4, 1.9) | 0.15 | 0.73 |
| V̇O₂ (ml·kg⁻¹·min⁻¹) | 13.7 (± 3.8) | 11 (± 1.4) | -2.7 ( -5.3, 0.05) | 0.93 | 0.04 |
| V̇O₂ (% predicted) | 57.3 (± 8.9) | 55 (± 5.9) | -2.3 ( -9.5, 5) | 0.30 | 0.53 |
| HR (bpm) | 106 (± 14) | 104 (± 13) | -2 ( -15, 10) | 0.17 | 0.70 |
| WR (W) | 53 (± 26) | 62 (± 21) | 9 ( -13, 31) | 0.38 | 0.39 |
| V̇E/V̇CO₂ slope | 36.2 (± 2.5) | 36.6 (± 2.5) | 0.4 ( -1.9, 2.7) | 0.17 | 0.70 |
| V̇E/V̇O₂ | 26.7 (± 2) | 26.3 (± 3.2) | -0.4 ( -2.9, 2.1) | 0.16 | 0.72 |
| V̇E/V̇CO₂ | 30.9 (± 2.1) | 30.9 (± 2.7) | 0 ( -2.3, 2.3) | 0.00 | 0.99 |
| VT2 |  |  |  |  |  |
| Time (min) | 10.4 (± 1.5) | 9.5 (± 1.9) | -1 ( -2.5, 0.6) | 0.57 | 0.22 |
| V̇O₂ (ml·kg⁻¹·min⁻¹) | 21.8 (± 6.6) | 16.9 (± 2.5) | -4.9 ( -9.6, -0.2) | 0.96 | 0.05 |
| V̇O₂ (% predicted) | 88.9 (± 5.6) | 84.1 (± 6.6) | -4.8 ( -10.5, 1) | 0.79 | 0.11 |
| HR (bpm) | 140 (± 17) | 133 (± 15) | -7 ( -22, 8) | 0.44 | 0.34 |
| WR (W) | 116 (± 57) | 125 (± 37) | 9 ( -35, 53) | 0.18 | 0.69 |
| V̇E/V̇CO₂ slope | 35.9 (± 2.3) | 37.1 (± 3.4) | 1.2 ( -1.6, 3.9) | 0.41 | 0.35 |
| V̇E/V̇O₂ | 33.3 (± 3.5) | 34 (± 4) | 0.7 ( -2.7, 4.2) | 0.19 | 0.66 |
| V̇E/V̇CO₂ | 31.6 (± 2.5) | 31.6 (± 3.4) | 0 ( -2.8, 2.7) | 0.01 | 0.98 |

CPET: cardiopulmonary exercise testing; HR: heart rate; OUES: oxygen uptake efficiency slope; RER: respiratory exchange ratio; WR: Work Ratio; VCO₂ = carbon dioxide output; VE: minute ventilation; V̇O₂: oxygen consumption; VT: ventilatory threshold. Data are mean ± SD. Group comparisons used Student’s t-test, and effect sizes are reported as Cohen’s d.

**Supplemental Table S2.** Correlations between myocardial work indices and CPET/vascular variables in Long COVID and Control Groups

| **Variables** | **Control (n=11)** | | |  | **Long COVID (n=10)** | | |
| --- | --- | --- | --- | --- | --- | --- | --- |
|  | ***p-*value** | **r** | **R^2^** |  | ***p-*value** | **r** | **R^2^** |
| Strain GLS (%) vs |  |  |  |  |  |  |  |
| V̇O₂ peak (ml.kg^-1^.min^-1^) | 0.97 | -0.01 | 0.0002 |  | 0.06 | -0.61 | 0.37 |
| GWW (mmHg%) vs |  |  |  |  |  |  |  |
| V̇E peak (L/min) | 0.49 | -0.23 | 0.05 |  | 0.06 | 0.61 | 0.37 |
| V̇O₂ peak (ml.kg^-1^.min^-1^) | 0.19 | -0.43 | 0.18 |  | 0.17 | 0.47 | 0.22 |
| GWE (%) vs |  |  |  |  |  |  |  |
| V̇E peak (L/min) | 0.42 | 0.27 | 0.07 |  | 0.05 | -0.63 | 0.39 |
| V̇O₂ peak, VT1 (ml.kg^-1^.min^-1^) | 0.08 | 0.55 | 0.30 |  | 0.93 | -0.03 | 0.001 |
| GWI (mmHg%) vs |  |  |  |  |  |  |  |
| VVM (L/min) | 0.57 | 0.19 | 0.04 |  | 0.08 | -0.58 | 0.34 |
| Shear rate max (s^-1^) | 0.10 | 0.51 | 0.26 |  | 0.14 | 0.50 | 0.25 |

Data are Pearson correlation *p*-values (*p*), correlation coefficients (r), and coefficients of determination (R²) for associations between myocardial strain and work indices (WI), BMI, and CPET variables in the Control (n = 11) and Long COVID (n = 10) groups. BMI: body mass index; CPET: cardiopulmonary exercise test; GLS: global longitudinal strain; GWE: global work efficiency; GWI: global work index; GWW: global wasted work; V̇CO₂: carbon dioxide output VE: minute ventilation; V̇O₂: oxygen consumption; VT1 and VT2: first and second ventilatory thresholds.

**Supplemental Table S3.** Brachial Artery Endothelial Function in Long COVID and Control Groups

| **Variables** | **Control**  **(n=11)** | **Long COVID**  **(n=10)** | **Mean Difference**  **(95% CI)** | **Cohen’s *d*** | ***p*-value** |
| --- | --- | --- | --- | --- | --- |
|  |  |  |  |  |  |
| Resting diameter (mm) | 3.53 ± 0.73 | 3.88 ± 0.75 | 0.35 ( -0.33, 1.02) | 0.47 | 0.29 |
| Peak diameter (mm) | 3.82 ± 0.78 | 4.11 ± 0.79 | 0.29 ( -0.42, 1.01) | 0.37 | 0.40 |
| Recovery (mm) | 3.56 ± 0.83 | 3.89 ± 0.74 | 0.34 ( -0.38, 1.06) | 0.43 | 0.34 |
| FMD (%) | 8.12 ± 1.16 | 5.15 ± 1.35 | -2.97 ( -4.15, -1.79) | 2.30 | <0.001 |
| FMD (mm) | 0.28 ± 0.06 | 0.23 ± 0.09 | -0.05 ( -0.13, 0.02) | 0.70 | 0.06 |
| FMD_R_ (%) | 8.1 ± 5.39 | 5.65 ± 4.97 | -2.45 ( -7.2, 2.31) | 0.47 | 0.29 |
| Rest shear Rate (s⁻¹) | 141 ± 122 | 170 ± 80 | 29 ( -66, 125) | 0.28 | 0.53 |
| Peak Shear Rate (s⁻¹) | 993 ± 471 | 578 ± 161 | -415 ( -743, -86) | 1.15 | 0.01 |
| AUCSR (s⁻¹) | 23622 ± 16514 | 11801 ± 6309 | -11822 ( -23475, -168) | 0.93 | 0.04 |
| AUC max (u.a) | 15457 ± 5472 | 7576 ± 3865 | -7881 ( -12251, -3511) | 1.65 | 0.001 |

FMD: flow-mediated dilation; FMD_R_: resting flow-mediated dilation; AUCSR: area under the curve of shear rate. Data are mean ± SD. Group comparisons used Student’s Group comparisons used Student’s t-test, and effect sizes are reported as Cohen’s d.
